# The Potential Role of the Proteases Cathepsin D and Cathepsin L in the Progression and Metastasis of Epithelial Ovarian Cancer

**DOI:** 10.3390/biom5043260

**Published:** 2015-11-20

**Authors:** Md Zahidul Islam Pranjol, Nicholas Gutowski, Michael Hannemann, Jacqueline Whatmore

**Affiliations:** 1 Institute of Biomedical and Clinical Science, University of Exeter Medical School, Exeter, Devon EX1 2LU, UK; E-Mails: Z.Pranjol@exeter.ac.uk (M.Z.I.P.); N.J.Gutowski@exeter.ac.uk (N.G.); 2 Royal Devon and Exeter NHS Foundation Trust, Exeter, Devon EX2 7JU, UK; E-Mail: michael.hannemann@nhs.net

**Keywords:** epithelial ovarian cancer, angiogenesis, metastasis, cathepsin D, cathepsin L

## Abstract

Epithelial ovarian cancer (EOC) is the leading cause of death from gynecologic malignancies and has a poor prognosis due to relatively unspecific early symptoms, and thus often advanced stage, metastasized cancer at presentation. Metastasis of EOC occurs primarily through the transcoelomic route whereby exfoliated tumor cells disseminate within the abdominal cavity, particularly to the omentum. Primary and metastatic tumor growth requires a pool of proangiogenic factors in the microenvironment which propagate new vasculature in the growing cancer. Recent evidence suggests that proangiogenic factors other than the widely known, potent angiogenic factor vascular endothelial growth factor may mediate growth and metastasis of ovarian cancer. In this review we examine the role of some of these alternative factors, specifically cathepsin D and cathepsin L.

## 1. Introduction

Epithelial ovarian carcinoma (EOC) is the leading cause of death among gynecological cancers in the western world. Worldwide, approximately 200,000 women are diagnosed with this malignancy, with 125,000 disease related deaths each year [1]. The high mortality of this disease can be explained by late diagnosis at an advanced disease state, with widespread metastasis within the peritoneal cavity [2]. Symptoms are vague at the primary stage, and hence early diagnosis is difficult. Approximately, 70% of patients are diagnosed with the International Federation of Gynecology and Obstetrics (FIGO) stage III or IV, with a poor five year survival rate. Although the ideal primary cytoreductive surgery and combination chemotherapy with platinum have improved the prognosis of patients with malignant ovarian cancer, the 5-year survival rate remains ~40% [3,4].

Approximately 90% of human ovarian cancers are thought to originate from the epithelium [5]. Initially, EOC cells tend to undergo epithelial-to-mesenchymal (EMT) transition which weakens the attachment of epithelial cells to the basement membrane and loosens the intercellular adhesion between neighboring epithelial cells. Loss of expression of E-cadherin correlates with EMT and the acquisition of an invasive phenotype, and cells with low E-cadherin expression have been found in ascites and at metastatic sites [6,7,8]. As a part of a feedback loop, other cadherins are upregulated in these cells e.g., N-cadherin and P-cadherin [9,10]. These transformed cells are shed as single cells or clusters into ascites or peritoneal fluid and spread through the peritoneum, especially to the omentum [11]. At the omentum, the tumor cells undergo mesenchymal-to-epithelial transition into an epithelial phenotype; a transformation that is vital to allow them to respond to paracrine growth factors and sustain rapid growth [11]. The disseminated cancer cells first interact with the mesothelial layer that covers the omentum. Alpha and beta integrins on both cancer and mesothelial cells have been shown to bind to each other, initiating attachment of cancer cells to the omentum [12,13]. This attachment induces upregulation of matrix metalloproteinases (MMPs) 2 and 9 in cancer cells that cleave the extracellular matrix proteins fibronectin, vitronectin and collagens [11]. Early metastasis is well-coordinated via adhesion and proteolysis, which provides a niche for EOCs to establish secondary foci in the omentum by invading the basement membrane.

In order for the secondary tumor to grow and survive, angiogenesis is vital. In the tumor microenvironment the balance between pro- and anti-angiogenic factors favors the proangiogenic process, leading to activation, proliferation and migration of the endothelial cells lining vasculature and excessive genesis of new blood vessels from the existing ones. To date, several proangiogenic factors have been identified to be secreted by EOCs which could potentially drive angiogenesis and metastasis; these include various isoforms of the major pro-angiogenic mediator vascular endothelial growth factor (VEGF) as well as angiopoietin-2, basic fibroblast growth factor (bFGF), heparin-binding EGF-like growth factors (hb-EGF), and cytokines such as interleukins 6 and 8, and transforming growth factor-β1 (TGFβ1) [14,15,16,17].

Although these factors are now known to be secreted from EOCs, their involvement in inducing metastatic angiogenesis in secondary locations such as the omentum is not fully understood. Indeed, recent evidence suggests that omental metastasis of EOC may primarily occur via non-VEGF dependent pathways [18]. Further to this observation, several other potential proangiogenic factors were identified to be secreted from EOCs. These included cathepsin D (CathD) and cathepsin L (CathL) which were shown to induce proangiogenic responses in disease-relevant human omental microvascular endothelial cells [18]. These data suggest that these proteases may play an important role as alternative mediators of metastasis of EOC to the omentum. Since the clinical prevention and treatment of this pathological event is so critical to patient well-being, understanding the function of alternative mechanisms of angiogenesis may be important in targeting future therapies. In this review we aim to summarize the biological and clinical significance of CathD and CathL, initially in physiological cell regulation and then as potential pro-tumorigenic factors regulating progression steps in both primary and metastatic tumors including cell detachment, proliferation and migration, apoptosis and angiogenesis. In particular we will summarize what is known about their role in the etiology of both primary and secondary EOCs.

## 2. Cathepsin D

CathD is a soluble lysosomal aspartic endopeptidase primarily involved in degrading unfolded or non-functional proteins intracellularly. The protein is synthesized in rough endoplasmic reticulum as inactive preprocathepsin D (43 kDa), which is in turn cleaved and glycosylated to form 52 kDa procathepsin D (pCathD) containing two N-linked oligosaccharides modified with mannose 6-phosphate (M6P) residues. pCathD is then targeted to intracellular vesicular structures such as lysosomes, endosomes and phagosomes both by M6P receptor (M6PR)-dependent and-independent pathways (reviewed in [19]). The latter pathway of targeting is not fully understood; however the sphingolipid activator precursor protein pro-saponin has been suggested to be involved [20].

Once pCathD enters the late endosome, the low pH induces its dissociation from M6PR and subsequently the phosphate group is removed. Proteolytic cleavage of propeptide (44aa) on pCathD generates active intermediate enzyme [21]. The propeptide (also known as activation peptide) is essential for the correct folding, activation and delivery of the protein to lysosomes [22,23]. This peptide, which is expressed in, and secreted from, cancer cells, has also been demonstrated to act as a growth factor for tumor cells [24]. The intermediate CathD is further processed by cysteine proteases and autocatalysis to generate mature CathD (48 kDa) containing a heavy chain (34 kDa) and a light chain (14 kDa) [25]. The optimum pH for CathD activity is 3.5 at which it is highly proteolytically active [26]. However, proteolytic activity has also been reported at neutral pH in the cytosol of apoptotic cells and in neurofibrillary degeneration [27,28].

### 2.1. Physiological Roles of CathD as Both AN Intracellular and Extracellular Protein

CathD has been shown to play a significant role during fetal development. The lysosomal system matures gradually which correlates with increased CathD levels in all tissues [29]. A reduction of CathD expression or its catalytic activity results in neurodegenerative disorders. CathD knockout mice die shortly after birth and display significant neurodegeneration [29]. Congenital mutations in the CathD gene lead to a reduction in expression and subsequent production of enzymatically inactive protein that results in typical neuronal ceroid lipofuscinoses in dogs and humans [30,31,32,33,34,35]. Recently, it has been shown that CathD deficiency is associated with Parkinson’s disease [36]. Interestingly, increased CathD expression and activity in cardiac cells induces heart failure in postpartum female mice [37]. Higher CathD levels have also been suggested to play an important role in the pathogenesis of autism [38].

Several physiological functions of CathD have been suggested based on its proteolytic activity to cleave structural and functional proteins and peptides. These include metabolic degradation of intracellular proteins, activation and degradation of polypeptide hormones and growth factors such as plasminogen, prolactin, endostatin, osteocalcin, thyroglobulin, insulin-like growth factor binding proteins (IGFBP) and secondary lymphoid tissue chemokine (SLC); activation of enzymatic precursors of CathL, CathB and transglutaminase 1; and processing of the enzyme activators and inhibitors prosaposin and cystatin C (reviewed in [19]).

Although CathD is a lysosomal enzyme and its enzyme activity is usually regulated within the acidic compartment of lysosomes, it has been shown to be enzymatically active and biologically relevant extra-lysosomally at cytosolic pH, for instance in the control of apoptosis as discussed later.

Unlike other aspartic endopeptidases, under normal physiological conditions, pCathD is sequestered to the lysosome and not secreted extracellularly. However, in some conditions, pCathD/CathD escape the normal targeting pathway and are secreted from the cells. Most probably, over-expression of pCathD saturates the limited number of M6PR binding sites available and the protein accumulates in the cytosol, and is subsequently secreted via an as yet unknown mechanism [39]. Indeed pCathD has been found in human, bovine and rat milk and serum and the presence of both pCathD and CathD (34 kDa) was observed in human eccrine sweat and urine [40,41,42,43]. CathD in human eccrine sweat was found to be proteolytically active at sweat pH 5.5 [44]. Interestingly, there is increasing evidence that extracellular CathD may act via both proteolytic dependent and independent mechanisms.

### 2.2. Expression of CathD in Ovarian Cancer

In many cancer microenvironments, pCathD is a major secreted protein. In the last 2 decades, studies have shown increased overexpression and hypersecretion of CathD in numerous cancer types including ovarian cancer, but also in breast cancer, endometrial cancer, lung cancer, malignant glioma, melanoma and prostate cancer (Table 1) [18,45,46,47,48,49,50,51,52,53,54,55,56,57,58]. Early studies investigating ovarian carcinoma suggested that the expression level of CathD was associated with increased cell differentiation and with histological type [59,60]. Additionally, numerous immunohistochemistry studies have indicated that enhanced CathD expression is an indicator of malignancy in serous ovarian cancer [61,62,63], for instance Losch *et al.* observed that over 70% of invasive ovarian cancers express CathD [62]. Intriguingly, however, it has also been shown that in ovarian tumors that do express CathD, a high expression level was associated with a favorable survival prognosis [63]. More recently, in an investigation into omental metastasis of ovarian cancer we have observed a significantly higher expression of CathD in the omental lesion of serous ovarian carcinoma compared with omentum from patients with benign ovarian cystadenoma and that high omental mesothelial expression of CathD was associated with poor disease-specific survival (DSS) [64]. This stronger expression of CathD in mesothelial cells was observed close to the metastatic tumor, suggesting a paracrine effect for factors secreted from the tumor cells contributing to the increased CathD expression.

A number of studies have also examined CathD expression in breast cancer. CathD overexpression is correlated with increased risk of clinical metastasis and short survival in breast cancer [45,46,47] and increased serum pCathD levels were detected in the plasma of patients with metastatic breast carcinoma [65]. Additionally, total CathD concentration in breast cancer tissue was much higher than in other tissues including normal mammary cells [66].

**Table 1:** Involvement of cathepsin D in the stages of tumor progression in different cancer types.

Cancer Type	Metastasis	Invasion	Angiogenesis	References
Breast	↑	↑	↑	[45,46,47,48]
Ovarian	ND	ND	↑	[18]
Prostate	↑	↑	↓	[49,50,51]
Endometrial	ND	↑	ND	[58]
Melanocytic	↑	↑	ND	[53]
Glioma	↑	↑	ND	[54]
Lung	ND	↑	ND	[57]

↑, increase in effects; ↓, reduction in effects; ND, not determined.

### 2.3. Role of CathD in Tumor Progression

It is now recognized that CathD has a potential role in multiple tumor progression steps, both in its intracellular and extracellular form.

As indicated above, a role for intracellular cytosolic CathD has been identified in apoptosis. Here the lysosomal enzyme is translocated to the cytosol due to lysosomal membrane permeabilization [28,67,68]. Subsequently, CathD actively cleaves the BH3-interacting domain (Bid) to form truncated Bid (tBid) which in turn triggers the insertion of Bax into the mitochondrial membrane [69,70], and leads to the release of cytochrome c from mitochondria into the cytosol [71]. Inhibition of enzymatically-active cytosolic CathD, using the inhibitor pepstatin A (pepA), partially delayed apoptosis induced by IFN-gamma or oxidative stress and when pepA was co-microinjected with CathD [68,69,70]. The role of CathD in inducing apoptosis has also been shown to be associated with caspases; the pan caspase inhibitor Z-VAD-FMK added in combination with pepA, induced a significant reduction in cell death compared to individual inhibitor treatments. This suggested a strong association between caspases and proteolytically active cytosolic CathD [72,73]. Additionally, CathD has been shown to cleave tau protein *in vitro* at pH 7 [27]. These studies suggest that, intracellularly, CathD is proteolytically active at cytosolic pH. However, this has been contested by other studies indicating that the effect of a mutant CathD, deprived of its catalytic activity, was indistinguishable from that of the normal enzyme [74,75].

Although, a role for intracellular CathD in apoptosis suggests that the protein may be anti-tumorigenic; this is in contrast to the functions observed for extracellular CathD.

**Figure 1:**
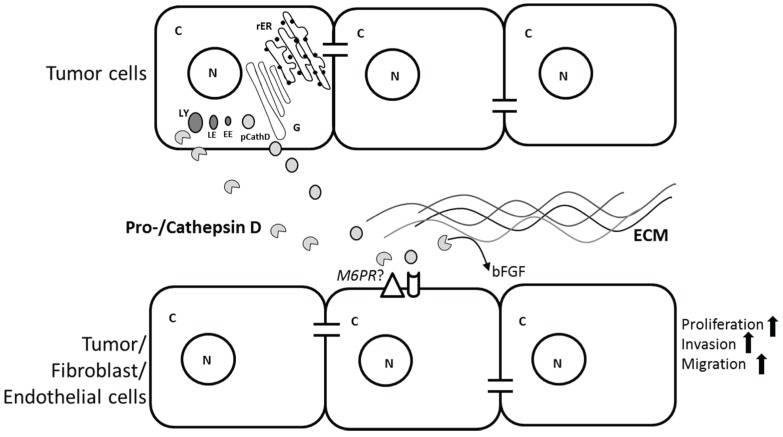
Potential roles of tumor cell-secreted procathepsin/cathepsin D (pCathD/CathD) on extracellular matrix (ECM), tumor, fibroblast and endothelial cells in the tumor microenvironment. pCathD is synthesized and processed in the rough endoplasmic reticulum (rER) and Golgi bodies (G), and subsequently transported to early endosome (EE), late endosome (LE) and finally lysosome (LY). Overexpressed pCathD/CathD is secreted into the extracellular space by tumor cells. Mature CathD cleaves ECM and releases basic fibroblast growth factor (bFGF) that may induce angiogenesis. Both pCathD and CathD induce tumor cell proliferation, and hence invasion via an autocrine mechanism. CathD induces proliferation of fibroblasts and migration of endothelial cells. Mannose-6-phosphate receptor (M6PR) may be involved in inducing the proliferative effects. C and N denote cytoplasm and nucleus, respectively.

For instance CathD is secreted by EOC cancer cell lines (SKOV3 and A2780) [18], is present in the ascites of patients suffering from ovarian cancer (unpublished data), and exogenous CathD induced migration of human omental microvascular endothelial cells; a key step in angiogenesis during omental metastasis [18]. In a separate study pCathD and CathD have been reported to induce proliferation and migration of cancer cells, fibroblasts and endothelial cells in both a proteolytic dependent and independent manner [76] (Figure 1). While secreted pCathD is generally considered to be proteolytically inactive, it has been proposed that the acidic pH in the tumor microenvironment promotes the conversion of pCathD into mature, biologically active CathD. This was supported by data indicating that pCathD, collected from tumor-conditioned media, became auto-activated if the pH was lowered and was subsequently able to degrade ECM proteins and release growth factors such as bFGF [48,77,78], steps important for cancer cells to invade surrounding tissue [79].

There is evidence that CathD may induce mitogenic responses via both proteolytic-dependent and—independent mechanisms. Both the wild type and mutant form of CathD were shown to induce fibroblast proliferation via a mechanism whereby they acted as a protein ligand [80]. In the latter study, the authors demonstrated an interaction between M6PR and pCathD. Co-incubation with excess M6P partially prevented fibroblast proliferation. Although an unknown receptor molecule has been suggested to be involved, the identity of this potential receptor has not yet been resolved.

CathD actions on tumor growth were further reported in studies showing that 3Y1-Ad12 rat tumor cells transfected with human CathD cDNA grew more rapidly at low or high cell densities *in vitro* and presented an increased experimental metastatic potential *in vivo* [81,82,83]. Additionally, both wild-type and mutated (Asn 231, proteolytically inactive) CathD stimulated proliferation of 3Y1-Ad12 cells embedded in Matrigel or collagen 1 matrices, colony formation in soft agar and tumor growth in athymic nude mice [84,85]. Again, an unknown receptor, other than M6PR, was suggested to be involved in CathD mediated cell growth as no inhibition of cell outgrowth was observed when excess M6P was added, suggesting that M6P did not compete with CathD interacting with M6PR. In the same study the propeptide (27–44aa) of pCathD was found not to be mitogenic, contradicting studies which found otherwise [24,66,86,87,88,89].

The role of CathD has also been extensively studied in human primary breast cancer. Upregulation of CathD expression was observed in estrogen receptor (ER) positive breast cancer cell lines treated with estrogen [90]. *In vitro* experiments with the MCF7 cell line supported these data and revealed that pCathD/CathD were overexpressed and hyper-secreted from these cells into the media. Further studies have reported that as a mitogen pCathD acts as a protein ligand rather than enzymatically and that purified pCathD from MCF-7 breast cancer cells stimulated MCF-7 cell growth on plastic via an autocrine mechanism [91]. Intriguingly, CathD has also been shown to selectively degrade macrophage inflammatory protein (MIP)-1α (CCL3), MIP-1β (CCL4), and SLC (CCL21) that, in turn, may affect the generation of the anti-tumoral immune response, the migration of human breast cancer cells, or both processes [92].

In recent years studies have emerged that suggest that CathD can induce angiogenesis *in vivo* and *in vitro.* *In vivo*, overexpression of CathD in xenografts in an athymic mice model correlated with increased vascular density. The number of microvessels was significantly increased by 1.5-fold and 1.9-fold in the CathD and CathD-Asn 231(proteolytically inactive) groups respectively, suggesting that CathD induces angiogenic effects via an unknown mechanism other than its proteolytic activity [85].

CathD has also been shown to induce blood vessel formation in the chick chorioallantoic membrane (CAM) model [93] and a role for CathD in angiogenesis was further illustrated by the observation that migration of human umbilical vein endothelial cells (HUVECs) and *in vitro* angiogenic tube formation were increased when cells were treated with active pure CathD. CathD was proteolytically active in these experiments as complete inhibition of angiogenesis, tube formation and migration was achieved by addition of pepA [93]. Proteolytically active CathD has also been suggested to induce angiogenesis in breast cancer by cleaving and releasing ECM-bound pro-angiogenic bFGF [48].

In contrast it has also been suggested that CathD activity may be anti-angiogenic For instance, pCathD secreted by prostate cancer cells was shown to have a possible role in generating angiostatin via proteolysis—a specific inhibitor of angiogenesis *in vitro* as well as *in vivo* [51].

## 3. Cathepsin L

CathL is a lysosomal ubiquitous cysteine proteinase that plays an important role in degrading endocytosed proteins as well as intracellular proteins [94,95]. CathL is translated as preprocathepsin L (ppCathL), processed into procathepsin L (pCathL) in the rough endoplasmic reticulum with a molecular mass of 30 kDa and a two-chain form with molecular masses 25 kDa and 5 kDa [96,97,98,99], and then transported to endosome/lysosomes in M6PR pathway [100]. It has been reported that 1, 10-phenanthrolin and pepstatin partially inhibited the processing of the proenzyme form of CathL to the mature enzyme and it has been speculated that metallo-proteinases or an aspartic protease such as CathD are involved in the proteolysis-mediated activation of CathL in the lysosome [101,102]. CathL can also be produced by autocatalysis of pCathL at pH 3 as demonstrated in an *in vitro* study of pCathL collected into conditioned medium of cultured murine fibroblasts [103].

### 3.1. Physiological Roles of CathL

The key functional role of CathL is to degrade proteins in lysosomes and it has been shown to be highly active at physiological pH 5.5–6 in lysosomes in several studies [103,104]. However, it is also known that CathL is secreted in different forms into the extracellular space in both physiological and pathological conditions, and retains its function as a protease. In physiology, CathL has been shown to degrade the Ii peptide of major histocompatibility complex II (MHC II) that in turn allows peptides derived from the proteolytic degradation of foreign or self-proteins to then bind to class II molecules and appear on the cell surface [105]. Interestingly, it has been shown that CathL is essential for MHC II mediated antigen presentation in cortical thymic epithelial cells but not in bone marrow-derived antigen-presenting cells *in vivo*. This was reflected in CathL deficient mice with a reduction in CD4+ T cells [105]. CathL has also been shown to degrade and process MHC II molecule-mediated antigen presentation [106]. CathL is essential for epidermal homeostasis and regular hair follicle morphogenesis and cycling; indeed, CathL-deficient mice develop periodic hair loss and epidermal hyperplasia, acanthosis, and hyperkeratosis [107].

It has also been reported that CathL null mice showed reduced bone mass compared to wild type mice suggesting a role for CathL in bone remodeling. CathL null mice showed significant reduction in bone volume in travecular, but not cortical, bone compared to wild type. Bone loss was exacerbated in null mice (compared to wild type) following ovariectomy suggesting that CathL is stimulated by external stimuli (e.g., estrogen) and is likely to play a role in controlling bone turnover during normal development and in pathological states [108].

### 3.2. Cathepsin L Secretion

A secreted form of CathL was first identified as a major secreted protein from a transformed mouse fibroblast cell line [103]. However, the mechanism of CathL secretion is still a mystery. pCathL has only one single chain carbohydrate, and hence it has low affinity for M6PR [109,110,111]. This observation suggests that not all CathL binds to M6PR and therefore is secreted from the cell by default protein trafficking [100]. M6PR saturation, downregulation or redistribution to the plasma membrane has also been suggested [112,113]. Interestingly, hypoxia was shown to induce secretion of CathL from the murine fibrosarcoma cell line KHT-LP1 which may accelerate the metastatic process in these cells [114]. CathL has also been shown to be protective against bacterial infection in airways of mice [115].

### 3.3. Expression of CathL in Ovarian Cancer

CathL has been linked to tumor invasion and metastasis, particularly by degrading ECM components such as proteoglycans, aggrecan, elastin, laminin, fibronectin and collagens I, II, IX, XI [116,117,118,119,120,121]. In ovarian cancer an increased level of secreted CathL was observed in the sera of epithelial malignant EOC patients compared to those with benign ovarian tumors and normal ovarian tissue [122,123]. These studies also showed that there was a significant increase in the tumor expression of CathL mRNA levels which correlated with its protein levels in serum. CathL has been suggested to be involved in the invasion and metastasis of EOC, and hence maybe a marker of advanced stage ovarian cancer [123]. This is supported by our own data demonstrating that the endothelium of vessels within omentum hosting metastatic ovarian high-grade serous carcinoma expressed significantly increased CathL *in vivo* compared with omentum from control patients with benign ovarian cystadenoma [64].

### 3.4. Role of CathL in Tumor Progression

CathL may play a role in the proliferation of ovarian cancer cells, although the data are contradictory. CathL had little effect on cell growth and proliferation of the A2780 ovarian cancer cell line [123], whereas downregulation of CathL significantly inhibited the proliferative and invasive capability of SKOV3 ovarian cancer cells [124].

Over-expression of CathL has been linked to metastasis following ras transformation of NIH/3T3 cells [125] and non-metastatic melanoma cells were converted to a metastatic state by over-expression of CathL [126]. An extra-lysosomal role for CathL has been suggested in human and murine melanoma cells in the context of metastasis [127]. Recently, it was shown that CathL is involved in B16F10 melanoma cell invasion, particularly through cell migratory influences. There was approximately a 70% reduction in CathL anti-sense clone invasion and migration compared to control after 24 h. However, when CathL-induced proliferation was tested in these cells, no difference was found between the rate of proliferation of antisense cell and control cell colonies. Overall, the results suggested that secreted CathL has direct effects on cell motility and contributes, via its proteolytic action, to the active invasion of melanoma cells [128]. CathL-induced pancreatic cancer cell invasion was also observed in RT2 mice. CathL null mice had a significant reduction in tumor volume and invasion, suggesting extracellular proteolytic activity. In contrast to the melanoma cell study discussed above, the latter work demonstrated significant proliferative effects of CathL on pancreatic cancer cells, with a 58% decrease in proliferation in CathL knockout cells [129].

Recently, CathL derived from skeletal muscle cells transfected with bFGF has been shown to promote migration of HUVECs [130]. Cell migration was examined in the presence of the cell impermeable CathL-proteolytic activity inhibitor Z-Phe-Tyr-Cho and CathL for 12 hours. The data revealed a significant reduction in HUVEC migration, suggesting that CathL influences cell migration in a manner dependent on its proteolytic activity. Subsequently, CathL was found to activate c-Jun N-terminal kinase (JNK) in migratory HUVECs [130]. However, the exact role of CathL in activating the JNK pathway has not been elucidated.

**Figure 2:**
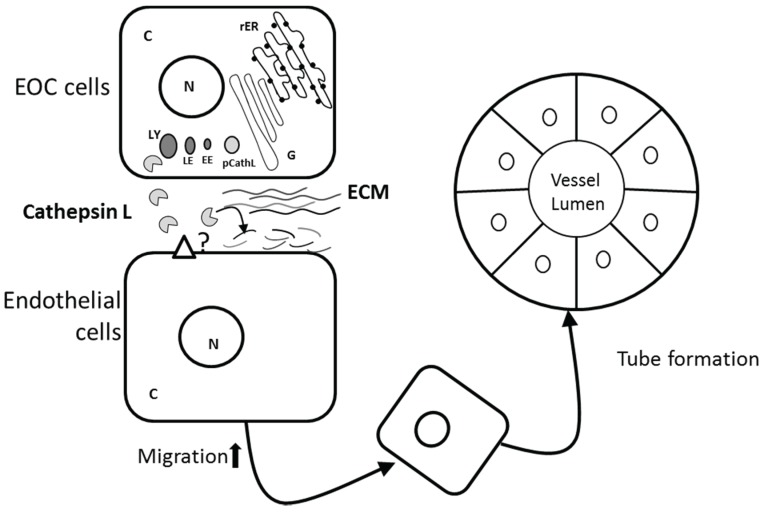
Potential proangiogenic role of cathepsin L in EOC. Procathepsin L (pCathL) is synthesised and processed in the rough endoplasmic reticulum (rER) and Golgi bodies (G), and subsequently translocated to early endosome (EE), late endosome (LE) and finally lysosome (LE). Mature CathL is secreted by epithelial ovarian cancer (EOC) cells and induces migration in endothelial cells via an unknown receptor. Tumor-secreted CathL may also degrade extra cellular matrix (ECM) components, facilitating new vessel formation.

Evidence for a role for CathL in angiogenesis is also contradictory. Recently SKOV3 and A2780 EOC cells were shown to secrete CathL. Exogenous addition of CathL to human omental microvascular endothelial cells used as an *in vitro* model of omental angiogenesis, induced migration and *in vitro* tube structure formation [18] (Figure 2). Together, these data suggest that CathL may trigger a proangiogenic phenotype in these endothelial cells.

In contrast to the pro-angiogenic role of CathL discussed above, both secreted and intracellular CathL has been shown to release endostatin, a potent inhibitor of angiogenesis, by cleaving ECM collagen [131]. Since the tumor microenvironment provides an acidic milieu, CathL can efficiently cleave collagen even outside the cells. However, in other studies, CathL had no effect on angiogenesis. For instance, Gocheva *et al.* (2006) demonstrated that CathL had no significant effects on microvascular density in pancreatic cancer in mice [129]. When evaluating the role of CathL in angiogenic switching in homozygous cathepsin knockout RT2 mice compared to control mice, the authors found that there was no significant effect on the development of these precursor lesions, suggesting that CathL did not contribute to angiogenic switching. However, other cathepsins, *i.e.* cathepsins B and S, were found to be very important in inducing angiogenesis in the same study [129].

Intriguingly, endothelial progenitor cells (EPCs) have been reported to produce CathL which in turn induces angiogenesis. Urbich *et al.* showed that EPCs were able to stimulate neovascularization and blood flow in the ischemic murine hind leg after injection into the affected leg [132]. These EPCs displayed significantly enhanced expression of CathL compared to mature endothelial cells as revealed by mRNA array analysis. It has been suggested that in the neovascularization process CathL activity may be extra- or pericellular. Indeed, mature CathL has been shown to maintain its proteolytic activity in the extracellular environment at neutral pH by the chaperone action of a p41 splice variant of the MHC class II-associated invariant chain [133], which also is strongly expressed in EPCs [132]. Such activity may facilitate EPC invasion and neovascularization and, interestingly, CathL deficient mice suffered from impaired neovascularization. Furthermore, mice treated with CathL-deficient bone marrow cells demonstrated a significant reduction in angiogenesis [132]. These data are supported by the observation that CathL expressed in EPCs cells plays a critical role in intraocular angiogenesis [134]. However, although CathL has been suggested to induce angiogenesis in these studies, its mechanism of action has not been elucidated.

## 4. Conclusions

Proteases such as CathD and CathL had long been known for their intracellular protein-degrading activities. However, a key role in cancer biology is now recognized, particularly their proteolytic function in ECM breakdown and thus facilitation of invasion. Interestingly, it is now becoming accepted that these proteases may also promote tumorigenesis and metastasis via non-proteolytic actions, although there is still a relative lack of understanding regarding their receptors and downstream intracellular effectors and signaling pathways. In EOC the urgent need to develop effective therapeutic approaches to improve patient outcomes highlights the importance of better understanding the role of key factors, such as the cathepsins, in driving ovarian tumor progression and metastasis in order to identify potential molecular therapeutic targets.
